# Promoting Mask Use on TikTok: Descriptive, Cross-sectional Study

**DOI:** 10.2196/26392

**Published:** 2021-02-12

**Authors:** Corey H Basch, Joseph Fera, Isabela Pierce, Charles E Basch

**Affiliations:** 1 Department of Public Health William Paterson University Wayne, NJ United States; 2 Department of Mathematics Lehman College The City University of New York Bronx, NY United States; 3 Department of Health and Behavior Studies Teachers College Columbia University New York, NY United States

**Keywords:** TikTok, COVID-19, social media, infodemiology, infoveillance, mask use, prevention, promotion, communication, public health, cross-sectional, content analysis, transmission

## Abstract

**Background:**

Over the past decade, there has been an increasing secular trend in the number of studies on social media and health.

**Objective:**

The purpose of this cross-sectional study was to examine the content and characteristics of TikTok videos that are related to an important aspect of community mitigation—the use of masks as a method for interrupting the transmission of SARS-CoV-2.

**Methods:**

In total, 100 trending videos with the hashtag #WearAMask (ie, a campaign on TikTok), along with 32 videos that were posted by the World Health Organization (WHO) and involved masks in any way (ie, all related WHO videos at the time of this study), were included in our sample. We collected the metadata of each post, and created content categories based on fact sheets that were provided by the WHO and the US Centers for Disease Control and Prevention. We used these fact sheets to code the characteristics of mask use.

**Results:**

Videos that were posted on TikTok and had the hashtag #WearAMask garnered almost 500 million views, and videos that were posted by the WHO garnered almost 57 million views. Although the ratio of the number of trending #WearAMask videos to the number of WHO videos was around 3:1, the #WearAMask videos received almost 10 times as many cumulative views as the WHO videos. In total, 68% (68/100) of the trending #WearAMask videos involved humor and garnered over 355 million cumulative views. However, only 9% (3/32) of the WHO videos involved humor. Furthermore, 27% (27/100) of the trending #WearAMask videos involved dance and garnered over 130 million cumulative views, whereas none of the WHO videos involved dance.

**Conclusions:**

This study is one of the first to describe how TikTok is being used to mitigate the community spread of COVID-19 by promoting mask use. Due to the platform’s incredible reach, TikTok has great potential in conveying important public health messages to various segments of the population.

## Introduction

As of November 30, 2020, there have been 62,363,527 COVID-19 cases and 1,456,687 deaths from COVID-19, and 13,082,877 cases and 263,946 deaths have been reported in the United States [1]. As with all emerging infectious disease outbreaks, public health messages have become centrally important. In fact, researchers have created infectious disease transmission models that are based on media influence–related data [2]. The sheer volume of information that is being generated throughout the COVID-19 pandemic has been classified as an “infodemic” by the World Health Organization (WHO) [3]. This influx of information (ie, correct information and incorrect/misleading information) can cause confusion, negate mitigation efforts [4], and result in serious negative consequences [5,6]. Members of the general public rely on different outlets for obtaining information. Recent studies have indicated that those who mostly rely on national news outlets believe that media coverage is largely accurate [7]. However, national news varies in content and focus; often presents information that is based on the different contexts of pandemics [8,9]; and at times, fails to capitalize on the opportunity to promote infection prevention techniques and coping strategies [8]. During the time when newspaper content was filled with information on COVID-19, popular social media outlets also began to provide information about the disease [10-14].

The majority of Americans who use the internet have, in part, done so to find web-based health information [15]. In a survey of US adults, 18% of participants (ie, in a sample of younger people who were generally unconcerned with political news) relied on social media as their customary source of news [15]. However, only 37% of these participants reported that they monitored news about the COVID-19 pandemic very closely, and over half (57%) of all participants reported that they have seen information about COVID-19 that seemed to be “completely made up” [15]. Social media is incredibly popular in the United States, as roughly 70% of the public has reported that they use some type of social media platform during their typical daily routines [16]. Prior to the COVID-19 pandemic, researchers raised questions about the proliferation of misinformation during public health emergencies [17-20], and one study on social media concluded that falsities spread more rapidly than the truth [21]. Several social media platforms have taken measures (eg, filtering posts from untrustworthy websites) for addressing concerns about the potentially harmful effects of misleading or incorrect information [22,23].

Public health professionals, agencies, and organizations have made efforts to use social media platforms to share information, or have partnered with popular members of these platforms to disseminate messages. One such effort has been promoting mask use on TikTok [24]. TikTok is a popular social media platform with approximately 800 million users worldwide and over 30 million users in the United States [25]. TikTok allows users to post 15-second or 60-second videos that are generally oriented toward entertainment. Roughly 42% of TikTok users are aged 18-24 years, and about 27% of frequent users are aged 13-17 years. As of November 12, 2020, 410 people in the United States aged between 15 and 24 years have died due to COVID-19 [26]. Although the incidence rate of such deaths is higher among people aged ≥25 years, studies have confirmed that younger individuals can transmit SARS-CoV-2 [27,28].

Over the past decade, there has been an increasing secular trend in the number of studies on social media and health, as indexed by the National Library of Medicine. Yet on December 5, 2020, a web-based search for the keyword “TikTok” only yielded 13 results, of which 4 were studies that focused on COVID-19. The purpose of this study was to examine the content and characteristics of TikTok videos that relate to an important aspect of community mitigation—the use of masks as a method for interrupting the transmission of SARS-CoV-2.

## Methods

The methods that we used in this study mirror those that were used in a prior study on TikTok and COVID-19 [10]. However, our study focused exclusively on the use of TikTok as an infection prevention tool for promoting mask use and mitigating the airborne transmission of SARS-CoV-2. On November 3, 2020, we identified 100 trending TikTok videos that used the hashtag #WearAMask, which is a campaign on TikTok that encourages mask use [29,30]. In addition, our sample included all WHO videos that mentioned masks (n=32). The WHO has approximately 2.7 million followers on TikTok; however, unlike the TikTok users in the #WearAMask campaign, the WHO’s followers are not represented by a hashtag. The reason our study was structured in this way was so that we could compare TikTok posts that used the most popular mask-promoting hashtag at the time of this study (ie, consumer-driven posts), with those from the most popular nongovernmental organization at the time of this study [31] (ie, professionally driven posts). Thus, the videos that we analyzed in this study represented consumer and professional interests for the same topic.

We conducted various quantitative data collection and analysis methods. Content categories were created by using fact sheets about mask use, which were provided by the WHO and the US Centers for Disease Control and Prevention [32,33]. Furthermore, we collected the metadata of each post, which included the date of the post; the number of views, likes, and comments; whether the post was in English or Spanish; the gender of the individual(s) in the video; whether the video included consumers (ie, members of the general public) or professionals (ie, doctors, registered nurses, public health professionals, etc); and whether the video involved dance, music, or humor. It is important to note that videos with the hashtag #WearAMask reported the exact number of views, while WHO videos reported a rounded number of views. Although these view counts were nuanced, we chose to report them as they appeared on TikTok, so as to not lose data. We also noted whether metadata characteristics or a mask appeared within the first 15 seconds of a video, whether the specific hashtag #WearAMask was mentioned or displayed in a video’s description, and whether an image of a mask or the word “mask” appeared in a video’s thumbnail. With regard to coding, we determined whether a video mentioned the following information: the hashtag #WearAMask, the correct use of a mask, the ramifications of improper mask use, guidelines for using fabric masks, the necessity of masks for people who do not practice social distancing, the importance of using a mask to prevent infection, three-layer masks, and comparisons among different types of masks. To establish interrater readability, one author (ie, IP) recorded the content of all included videos, and another author (ie, CHB) coded the content of a random sample of 20 videos. The agreement of the coded data was excellent (κ=0.98). Data analysis involved the calculation of descriptive statistics, which included frequencies and percentages. Since this study did not involve human subjects, it was not reviewed by the institutional review board at William Paterson University, as per their protocol. This study was also deemed exempt for review by the Teachers College, Columbia University Institutional Review Board.

## Results

A total of 132 TikTok videos were analyzed in this study. Of these 132 videos, 100 (75.8%) used the hashtag #WearAMask and garnered 494,824,395 views, and 32 (24.2%) were posted by the WHO and garnered almost 57 million views. Although the ratio of the number of trending #WearAMask videos to the number of WHO videos was 3:1, the #WearAMask videos received almost 10 times as many cumulative views as the WHO videos.

A total of 68% (68/100) of the trending #WearAMask videos involved humor and garnered over 355 million cumulative views, but only 9% (3/32) of the WHO videos involved humor. Additionally, while 27% (27/100) of the trending #WearAMask videos involved dance and garnered over 130 million cumulative views, none of the WHO videos involved dance. With regard to the trending #WearAMask videos, the proportion of videos was generally consistent with the proportion of cumulative views. With regard to the WHO videos, there were several notable differences. For example, while proper mask use was shown in 8 of the 32 WHO videos (25%), these videos received more than 75% (43,148,900/56,874,200, 75.87%) of the cumulative views. Additionally, while 15 of the 32 WHO videos (47%) mentioned wearing a mask, these videos received over 82% (47,049,700/56,874,200, 82.73%) of the cumulative views. In contrast, while 22 of the 32 WHO videos (69%) used music, these videos received less than 20% (10,534,400/56,874,200, 18.52%) of the cumulative views. Furthermore, although almost half of the WHO videos (15/32, 47%) mentioned that masks were essential infection prevention tools and garnered over 44 million views, this was not mentioned in a great majority of trending #WearAMask videos (Table 1).

Although our observations show how the presence of certain characteristics was associated with the number of cumulative views, independent 1-tailed *t* tests (α=.05) did not indicate whether these characteristics (ie, the use of dance, the use of music, mentions of wearing a mask, mentions of proper mask use, and mentions of masks as an essential infection prevention tool) led to higher view count averages. The tests we conducted for all videos and video categories returned *P* values of >.05. The presence of humor however had a marginal statistical effect *(P=.*052); videos that involved humor had an average view count of 5,010,849, while those that did not involve humor had an average view count of 3,211,939.

Of the 132 videos, 2 (1.5%) were recorded in English, 1 (0.8%) was recorded in Spanish, and 1 (0.8%) was recorded in both English and Spanish. The video that was recorded in Spanish had 4,600,000 views, and the video that was recorded in both English and Spanish had 3,500,000 views. Overall, 40.9% (54/132) of the videos only featured females, 32.6% (43/132) only featured males, and 16.7% (22/132) featured both.

Videos that used humor collectively received over 70% (66,329,979/92,922,611; 71.38%) of the total likes and over 75% (683,822/897,252; 76.21%) of the total comments.

Furthermore, although almost half of the WHO videos (15/32, 47%) mentioned that masks were essential infection prevention tools and garnered over 44 million views, this was not mentioned in a great majority (16/100, 16%) of trending #WearAMask videos (Table 1.)

**Table 1:** Observed characteristics, content, and view count of 132 TikTok videos (ie, 100 videos with the #WearAMask hashtag and 32 videos created by the World Health Organization).

Variables		#WearAMask videos (n=100)			World Health Organization videos (n=32)	
		Number of videos, n	Number of views, n (%)	Number of videos, n		Number of views, n (%)
Total		100	494,824,395 (89.69)^a^	32		56,874,200 (10.31)^a^
**Video characteristics**						
	Used dance	27	130,480,395 (26.37)	0		0 (0)
	Used music	58	284,671,795 (57.53)	22		10,534,400 (18.52)
	Used humor	68	355,304,595 (71.8)	3		465,700 (0.82)
	Mentioned or showed a mask in the first 15 seconds	59	240,836,695 (48.67)	27		55,412,700 (97.43)
	Mentioned #WearAMask in the video’s description	100	494,824,395 (100)	8		938,100 (1.65)
	Contained the word “mask,” an image of a mask, or an image of a person wearing a mask in the thumbnail	42	161,267,595 (32.59)	21		46,288,200 (81.39)
**Video content**						
	Used the term “wear a mask”	10	31,583,695 (6.38)	15		47,049,700 (82.73)
	Proper mask use	11	58,226,495 (11.77)	8		43,148,900 (75.87)
	Improper mask use	4	9,974,400 (2.02)	1		84,000 (0.15)
	Fabric mask use guidelines	0	0 (0)	6		1,265,100 (2.22)
	Wearing a mask when not social distancing	0	0 (0)	7		1,698,900 (2.99)
	Wearing a mask as an essential infection prevention tool	16	52,325,695 (10.57)	15		44,652,500 (78.51)
	Mentioned three-layer fabric masks	0	0 (0)	5		1,098,800 (1.93)
	Compared different types of masks	1	10,300,000 (2.09)	1		247,100 (0.43)

^a^This percentage refers to the number of views out of the total number of views for all 132 videos (N=551,998,595).

## Discussion

The 100 trending TikTok videos on mask use that were reviewed in this study were collectively viewed almost 500 million times, which indicates the immense popularity of this emerging social media platform. Although the ratio of the number of #WearAMask videos to the number of WHO videos was 3:1, the former received almost 10 times as many views as the latter. A relatively small number of videos addressed proper versus improper mask use, the necessity of wearing a mask when not practicing social distancing, or the importance of masks as an infection prevention tool.

The differences between the #WearAMask and WHO videos in terms of video format (ie, the use of dance, music, and humor) and the commensurate differences in number of views suggest that these video formats warrant consideration as an effective method for disseminating up-to-date, mask use–related messages to the younger population.

To date, there is a paucity of research on public health information that is conveyed on TikTok. Although younger populations do not seem to be the most vulnerable to developing COVID-19 or experiencing the harmful consequences of the disease, they play an important role in disease transmission [27,28]. It is therefore imperative that public health professionals find methods for communicating effectively with younger populations when it comes to disseminating information about community mitigation (eg, promoting mask use).

Despite the large, relative differences between #WearAMask and WHO videos in terms of the number of views, the fact that the WHO videos on mask use garnered almost 57 million views is encouraging. The #WearAMask videos clearly used a different approach from that of the WHO videos; the #WearAMask videos were more likely to use dance, music and humor. They were also far more likely to attract viewers. Research is needed to help inform the WHO and other public health agencies about methods for adapting their communications, which are generally about very serious subjects; and to appeal to their intended audiences. For example, the presence of entertainers has been associated with the popularity of messages on other social media platforms [34], and the presence of influencers in messages related to health has been increasing [35,36]. This suggests that social media can be an effective platform for disseminating public health messages [37]. However, to date, TikTok videos on mask use do not, for the most part, highlight masks as an essential infection prevention tool or demonstrate proper mask use. Given the short length of TikTok videos, further research is needed to determine the types of messages that can effectively be conveyed on this platform.

This study had several limitations and delimitations. First, the content on TikTok fluctuates on a constant basis, and our study was cross-sectional in design. Therefore, we could not generalize our findings to different time periods. Second, the sample of videos that we analyzed was relatively small, given the large volume of posts on TikTok. Third, we could not distinguish the number views from the number of viewers, as we did not know whether the same users viewed a video multiple times. Fourth, we could not determine whether the videos were viewed in their entirety. Furthermore, this study was delimited to focus specifically on masks, which are an effective infection prevention tool [38,39] given the airborne nature of SARS-CoV-2 (ie, the virus that causes COVID-19) [40,41]. However, there are other hashtags that also promote mask use. We selected #WearAMask because it had the largest number of collective views (ie, about 4.5 billion views at the time the data was coded) [29]. There are also other organizations that have posted videos on TikTok; however, the WHO has been noted as the most popular [31].

Despite these limitations and delimitations, this study is one of the first to describe how TikTok is being used to mitigate the community spread of COVID-19 by promoting mask use. The nature of this medium presents challenges for conveying complex information. Nevertheless, because of its widespread reach, TikTok has great potential in conveying important public health messages to various segments of the population. Future research is needed to develop methods for applying the characteristics of highly viewed videos to public health messages. This is necessary for helping people make informed decisions about health promotion and disease prevention in general, and COVID-19–related mask use in particular.

## Abbreviations

WHO: World Health Organization
